# Oral health screening for sport

**DOI:** 10.1038/s41415-025-9005-8

**Published:** 2026-02-27

**Authors:** Julie Gallagher, Ian Needleman, Paul Ashley, Peter Fine

**Affiliations:** University College London, Eastman Dental Institute, Centre for Oral Health and Performance, Bloomsbury Campus, Rockefeller Building, 21 University Street, London, WC1E 6DE, United Kingdom

## Abstract

Oral health is an essential component of general health and wellbeing, but there are many potential challenges to the oral health of athletes, including nutrition and hydration, exercise-induced immune suppression, lack of awareness, negative health behaviours and lack of prioritisation. Oral diseases should be preventable, and there are simple interventions that have good evidence of efficacy. Screening that allows early recognition of disease is well-established in healthcare as the first step in prevention of disease and promotion of positive health behaviours. This paper aims to provide an overview of the oral health problems that can be screened for in sport.

## Key points

- Provides a comprehensive guide to oral health screening for athletes.
- Describes the clinical indices for recording dental caries, erosive toothwear, periodontal diseases and orofacial trauma.
- Describes a useful questionnaire to measure impacts on athlete performance and wellbeing.
- Describes a useful questionnaire to measure oral health behaviours.

## Introduction

Oral health screening for elite athletes has been advocated for, as participation in sport appears to increase the risk of oral health problems.^1^^,^^2^^,^^3^ However, oral health screening is not yet routinely included in the athlete healthcare package. This paper aims to provide sports dentists with confidence to engage in sport-related oral health screening.

## Why is oral health screening needed in sport?

Oral health is an essential component of general health and wellbeing,^4^ but there are many potential challenges to the oral health of athletes, including sports nutrition, hydration and supplements, exercise-induced immune suppression, lack of awareness, negative health behaviours, and lack of prioritisation.^1^ Although many athletes will attend a dentist on a regular basis, many others report not having attended in the previous year.^2^^,^^5^^,^^6^ A survey of elite athletes preparing for the 2016 Rio Olympic Games found that fewer than half (39.5%) said they had attended for a dental visit within the previous six months.^7^ Those involved in athlete welfare should be aware of the lifetime burden of treatment need and its effect on quality of life of athletes,^8^ but it is also important for athletes to understand the possible oral health risks of an athletic lifestyle and feel empowered to adopt strategies to mitigate those risks.^8^^,^^9^

To compete successfully, at any level of sport, participants need to be well-trained, injury-free and healthy.^6^ Elite athletes, in particular, differ from the general population due to their greater levels of physical function, psychological function and perceived health.^10^ They rely on physical activity for their livelihood; therefore, the consequences of injury and illness are considerable.^10^ Elite athletes with oral health problems have consistently reported associated impacts on outcome measures, such as wellbeing, quality of life, training and performance.^2^^,^^5^^,^^6^ Problems such as acute dental infections or orofacial trauma can lead to time lost from training or competition but are reported infrequently.^6^ However, psychosocial impacts, including athlete-reported difficulties with eating, drinking, sleeping, relaxing and confidence, which have the potential to affect training quality, are more reported more often.^6^ For elite athletes, where winning comes down to the narrowest of margins, such impacts may have important consequences. Many sub-elite and recreational sport participants take their personal achievements just as seriously.

It is therefore important that athlete wellbeing is protected through prevention of injury and illness, including oral health problems.^11^ Oral diseases can be prevented with simple interventions that have been proven to be effective. Screening that allows early recognition of disease is well-established in healthcare as the first step in prevention of disease and promotion of positive health behaviours.

## What are the recommended approaches to oral health screening in elite sport?

As part of their pre-season preparation, elite athletes routinely undergo a periodic health examination (PHE) and the inclusion of regular oral health evaluation was recommended by the International Olympic Committee as far back as 2009.^12^ The PHE aims to assess each athlete's current health status and identify their risk of future injury or disease.^12^ For many elite athletes, the PHE may be their initial contact with medical (and/or dental) personnel, and can also provide an opportunity for brief preventive interventions.^12^ Best practice in elite sport should filter down through all levels and this could encourage regular oral health checks related to the sporting environment.

The PHE should ideally take place with enough time to manage any injuries, or medical or oral health problems well before the competition season starts.^3^^,^^12^ Furthermore, oral healthcare should be provided on a continuous basis and not, for example, just in the run up to and during major competitions.^5^

The United Kingdom National Screening Committee framework for screening includes four principles:^3^

- ‘Screening should improve health and wellbeing, and the benefits should outweigh the harms'^13^
- ‘The people being screened should be treated with respect'^13^
- ‘Screening should be available and accessible to everyone'^13^
- ‘Screening should represent a fair and proportionate use of public resources'.^13^

Oral health fits very well with the ethical criteria established by Wilson and Junger (1968)^14^ for a screening programme:

- ‘The condition should be an important health problem'^14^
- ‘The natural history of the condition should be understood'^14^
- ‘There should be a recognisable latent or early symptomatic stage'^14^
- ‘There should be a test that is easy to perform and interpret, acceptable, accurate, reliable, sensitive and specific'.^14^

The aetiology of dental caries, erosive tooth wear and periodontal diseases are well-understood, the clinical procedure associated with dental screening is familiar, and there are better outcomes for early intervention compared with standard care after symptoms occur. There are well-established clinical indices that are familiar to dentists, and these allow for detection of disease at an early stage. Many use a number system to link to risk and treatment need, making feedback simple. Routine radiographs for screening are not considered appropriate without a specific clinical indication.^15^ However, athletes should be referred for radiographs if there has been an interval of greater than two years since the most recent dental visit.^15^

Athletes are a group who could benefit from an oral health screening programme, carried out at their place of training.^16^ Many athletes ignore symptoms associated with overuse injuries and continue to train and compete despite the presence of pain and reduced function;^17^ therefore, it is not surprising if they also disregard symptoms associated with oral health problems. When asked, two-thirds (62.1%) of elite and professional athletes reported that convenience was the most important factor to consider when arranging dental visits.^7^ In one research project, where oral health screening was offered to elite and professional athletes, eight out of ten attended.^6^ The PHE often takes place for a whole cohort at an athlete training venue and comprises a series of individual examinations/screening tests. It is therefore entirely possible for a relatively rapid oral health screening to be included.

## Oral health screening

All clinical oral health screening should include examination of the mucosa for lesions in the palate, lips, tongue, throat/neck and cheeks. Other items include dental trauma, third molar problems, temporomandibular function and occlusion. Validated clinical indices such as the International Caries Detection and Assessment System,^18^ Pulp, Ulcer, Fistula, Abscess Index (PUFA),^19^ Basic Erosive Wear Examination (BEWE)^20^ and the Basic Periodontal Examination (BPE)^21^ provide clear guidance on their use and should ensure consistency between dentists when providing athlete feedback. We have previously also recommended their use as a core-outcome set for research.^22^

A questionnaire may also be useful to elicit further information from the athlete's perspective, and we provide an example of one, along with an example clinical data capture form, in our Oral Health Screening Toolkit.^23^

### Dental trauma

Current or historical trauma to teeth and the cause (sport related or not) should be recorded along with any soft tissue injury. The Andreasen Classification of Traumatic Dental Injury has been accepted within the International Classification of Diseases, endorsed by the World Health Organization,^24^ and the Eden-Baysal Index^25^ is a new five-digit index that has the potential to be useful in recording all relevant information.

### Third molar status

Problems such as potential for pericoronitis can be assessed as presence or absence of clinical features, including swelling and inflammation associated with a partially erupted third molar.

### Temporomandibular function

The temporomandibular joint should be assessed for function and pain. Temporomandibular pain/dysfunction may be a symptom of stress that athletes may disregard or omit to mention to their healthcare team.

### Dental caries

The International Caries Detection and Assessment System (ICDAS)^18^ is integral to the International Caries Classification and Management System (ICCMS) which links caries risk to preventive management.^26^ It allows for caries to be recorded from the initial stage, through to established lesions, that may still respond to preventive measures, and extensive lesions that require operative intervention. It was developed for use in dental education, clinical practice, dental research, and dental public health and comprises a two-digit system, where the first digit records the restorative status and the second digit caries status.^18^ The restoration and caries status are recorded for each surface and this can be combined to provide the caries experience at tooth level if preferred. There are additional specific codes to record teeth extracted for caries or orthodontics and unerupted teeth^18^ The ICCMS website provides a full description of the system.^26^

### Oral sepsis

The PUFA Index^19^ was developed as an epidemiological tool to measure and record the consequences of caries.^17^ The number of lesions is usually scored as 0, 1 and >2.^19^

### Erosive tooth wear

Details of how to use the BEWE have been published elsewhere.^20^ Each surface of each tooth in each sextant is examined and the worst score in each sextant is recorded.^20^ The total for the whole mouth is the usual value reported. This is then linked to severity and treatment need. In our research with elite athletes, we have considered a combined score of seven or more to indicate ‘increased risk'.^6^ Appropriate questioning of the athlete may be the first step in identifying disordered eating.^3^

### Periodontal diseases

The BPE^21^ was designed to provide a quick and simple screening tool for use in clinical practice and is linked specifically to the level of further examination required and treatment need.^21^ All dentists in the United Kingdom will be familiar with this index. A full description is published elsewhere.^21^

### Athlete-reported outcomes and outcome measures

As well as clinical oral health measures, self-reported perceptions can be useful.^6^^,^^10^ Factors likely to be most relevant for athletes are impacts from oral health problems on eating and drinking, sleeping and relaxing, and impact on confidence. Three items from the oral impacts on daily performance outcome measure^27^ used in the Adult Dental Health Survey of 2009^27^ can be used to assess these ‘psychosocial impacts'.^6^^,^^9^ A reference time frame of more than one year is unlikely to be relevant.^28^ The questions and response options are detailed in our Oral Health Screening Toolkit.^23^

The Oslo Sport Trauma Research Centre Overuse Injuries Tool^17^ consists of a set of four questions that have been modified and used to evaluate the impact of oral health problems on athletic performance in previous research.^6^^,^^9^^,^^28^ The questions and response options for oral health can be found in our Oral Health Screening Toolkit.^23^

### Oral health behaviours and risks to oral health

An athlete's lifestyle including usual diet and use of sports nutrition products may increase the risk of oral diseases^1^^,^^5^^,^^7^ and it may not be possible to alter these factors. It is, however, possible to mitigate these risks through athlete adherence to excellent oral health behaviours, the most important of which are effective daily plaque removal and use of fluoride toothpaste.^29^ The following information could be useful:^23^

- Time since last visit to the dentist
- Toothbrushing frequency
- Interdental cleaning (floss or interdental brushes) frequency
- Specific questions on diet should distinguish between in-training nutrition and out-of-training nutrition. Targeted questions on known risk factors, such as sports drink usage, could be considered.
- Use of a mouthguard (preferably custom-made) is recommended for athletes participating in contact sports^30^
- Tobacco use, vaping, alcohol consumption.

This list is not comprehensive and there may be clinical signs or symptoms that prompt further questioning.

## Providing information on individual susceptibility to disease

Athletes with significant clinical findings, such as trauma, caries, periodontitis, or oral sepsis, should be referred for appropriate further diagnostic tests, such as radiographs, and treatment. Athletes with early clinical signs of disease should be referred for preventive advice. At the oral health screening, brief oral health advice can be given to each athlete with their personalised feedback^3^^,^^9^ and a personalised oral health report provided, preferably with links to appropriate preventive actions. Athletes seem receptive to the use of the numbers generated by ICDAS, BEWE and BPE as a measure of severity of the disease identified. Digital intra-oral photographs are useful for documentation and as part of the feedback process.^9^ However, it is important to emphasise that susceptibility does not inevitably mean that individual athletes will experience disease, but rather that they should take special care to mitigate the risk through engaging in healthy behaviours.^31^ A traffic light system (Table 1) may be helpful when conveying individual risk. Our research has demonstrated that athlete oral health behaviours can be enhanced following brief interventions.^9^

**Table 1:** Suggested thresholds for athlete feedback

**Red**	**Amber**	**Green**
Established oral health problem requiring referral for further diagnostic tests and treatment	Early/reversible disease Requiring personalised preventive advice	Excellent oral health
Any BPE score 3, 4 Any ICDAS score 3, 4, 5, 6 Any BEWE score >3 or Total BEWE score > 7	Any BPE score 1, 2 Any ICDAS score 1, 2 Any BEWE score 2 or Total BEWE score 6	BPE score 0 ICDAS score 0 BEWE score 0,1
BEWE, Basic Erosive Wear Examination; BPE, Basic Periodontal Examination; ICDAS, International Caries Detection and Assessment System		

## Practical considerations for screening athletes

We have found that athletes and their support team appreciate the convenience of oral health screening provided at athlete training venues.^6^^,^^9^ If the screening is undertaken away from a dental surgery, then a suitable room at the facility is required to ensure privacy and confidentiality for each athlete.^6^^,^^22^

**Fig. 1:**
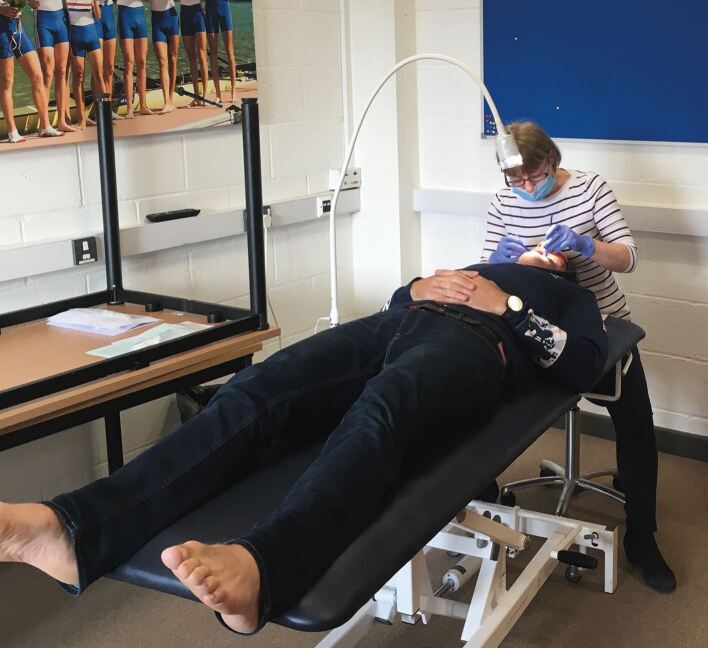
Example of oral health screening set up

As if in the dental surgery, athletes should be examined in a fully supine position, and the dentist and assistant should be seated comfortably.^6^^,^^22^^,^^23^ A treatment bench is usually available if physiotherapy or other medical services are provided at the venue. Otherwise, a portable massage table can be brought.^6^^,^^22^^,^^23^ Adequate illumination is essential and can be provided from a mobile examination lamp (e.g., DARAY X100LED, UK) or head torch.^6^^,^^22^^,^^23^ Figure 1 shows an oral health screening set up at a training venue. Ideally, compressed air from a portable dental unit would be used to dry the teeth (e.g., PDU II Standard, QDent, UK), otherwise cotton wool rolls may be used.^22^^,^^23^

A high standard of infection control is essential. Surfaces should be disinfected and personal protective equipment changed between each athlete examination.^6^^,^^22^^,^^23^ A fresh set of sterile single-use instruments should be used for each athlete and protective eyewear should be provided for the athletes during examination.^6^^,^^22^^,^^23^

## Behaviour change to enhance oral health

The *Delivering Better Oral Health* document provides clear guidance for the dental team regarding preventive advice, along with information about the level of certainty of the evidence base for the efficacy of simple preventive and oral health promotion behaviours.^32^ However, these interventions can only be effective if the recommended behaviour is adopted by individuals, and consistently adhered to in the real-life situation. In other words, it only works if you actually do it. For athletes, preventive advice can be provided in the form of ‘oral health drills'. Table 2 provides an example of suggested oral health drills. Behaviour change interventions, guided by contemporary behaviour change theory, such as the COM-B (capability, opportunity, motivation) model,^33^ may have an increased change of being effective.^32^

**Table 2:** Oral health drills

**Twice daily**	Brush using a manual or powered toothbrush, especially last thing at night
	Use toothpaste containing 1,350–1,500 ppm fluoride
	Your dentist may recommend fluoride toothpaste 5,000 ppm
	Spit after brushing – don't rinse
**Daily**	Before brushing, clean between your teeth as instructed
	Use a fluoride mouthwash at a different time to brushing
	Maintain a good diet for general health and oral health
	Avoid sugars other than for training and competition
**At least yearly**	Visit your dentist for regular oral health coaching and checks, particularly pre-season

Oral health screening at the regular training venue, or a training camp, may provide an opportunity to provide an educational session where the whole team can be given information about oral health and effective oral hygiene measures. Our feasibility study,^9^ conducted with three different elite athlete groups, combined oral screening with just such an intervention, and feedback from both athletes and support team members was positive.

## Summary

This paper has provided an overview of the oral health problems that can be screened for in sports participants. The recommended clinical indices can be used to identify oral conditions from an early stage, allowing clear feedback to athletes and their support teams, along with referral for further diagnostic tests as required. The suggested set of questions can help to assess the performance impacts of oral health problems. We have also provided suggestions for appropriate questions to screen athletes for behavioural factors that may increase or reduce their risk of oral health problems. Regular oral health screening, particularly in conjunction with sport-specific oral health promotion, has the potential to reinforce the importance of oral health within athlete general health, wellbeing and performance.

Oral health screening programmes need not be limited to elite groups, and screening conducted at sporting training venues has the potential to reach out to all groups in society with an interest in sport. Athletes should be aware that excellent oral health contributes to overall athlete health and wellbeing, and improves training adaptations, reduces the risk of injury and illness, and enhances competitive performance.
